# Supplementary material for: Interactions and pattern formation in a macroscopic magnetocapillary SALR system of mermaid cereal
Source: Nat Commun. 2024 Jun 27;15:5466. doi: 10.1038/s41467-024-49754-4 (PMC11211465; doi:10.1038/s41467-024-49754-4)
Supplement: Supplementary file 1 — Supplementary Information [file 41467_2024_49754_MOESM1_ESM.pdf]

Supplementary Information for "Interactions and Pattern Formation in a Macroscopic Magnetocapillary SALR System of Mermaid Cereal"

Supplementary Notes

Supplementary Note 1: Equilibrium spacing: model vs. experiments

Figure S1 shows the comparison of l_{eq} for varying m, a, and M between the experimental measurements and the model predictions in the mermaid regime.

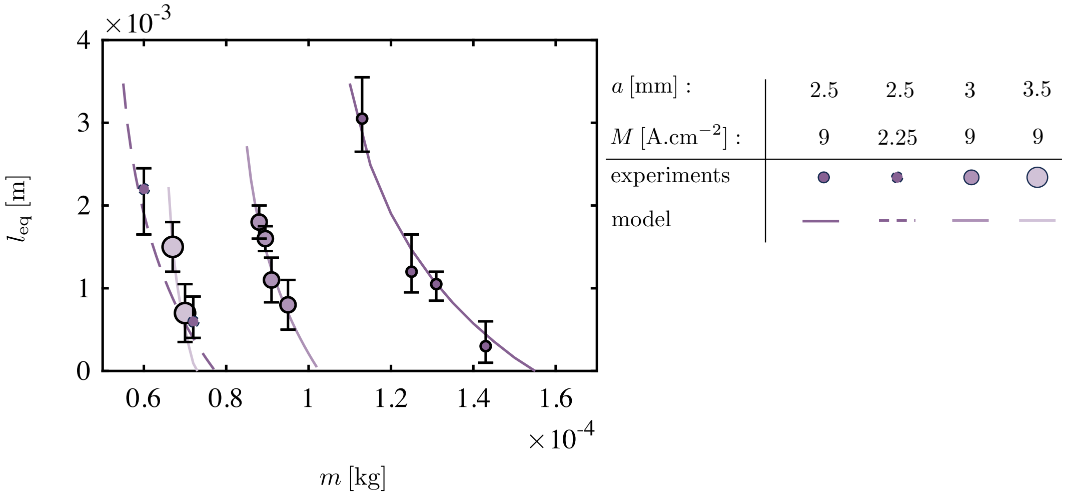

Figure S1: Comparison between experimental measurements (points) and prediction from model (curves) for l_{eq} in the mermaid regime. The error bars correspond to the min and max of the measured spacing over at least 3 independent trials.

Supplementary Note 2: Dimensional parameters in phase diagram

Table S1 shows the list of the three parameters controlled in the experiments to vary Bo and $\mathcal{M}$, namely disk radius a (0.05 mm tolerance), magnetic dipole strength M (10% tolerance), and disk mass m (10^{-3} g tolerance). Also, the measured experimental equilibrium length l_{eq} is indicated, with 0 corresponding to the Cheerios regime and ∞ corresponding to the fully repulsive regime. Finite values correspond to the mermaid regime.

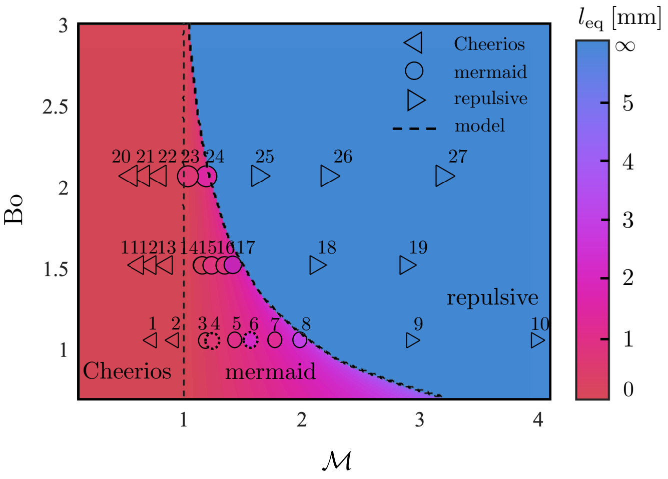

	a [mm]	M [A.cm^2]	m [$10^{-1} \times$ g]	l_{eq} [mm]
1	2.5	9	1.4	0
2	2.5	9	1.25	0
3	2.5	9	1.12	0.3 ± 0.25
4	2.5	2.25	0.72	0.6 ± 0.3
5	2.5	9	1.01	1 ± 0.1
6	2.5	2.25	0.64	2.2 ± 0.35
7	2.5	9	0.9	1.2 ± 0.3
8	2.5	9	0.85	3.05 ± 0.45
9	2.5	9	0.72	∞
10	2.5	9	0.61	∞
11	3	9	1.3	0
12	3	9	1.2	0
13	3	9	1.1	0
14	3	9	0.95	0.8 ± 0.3
15	3	9	0.92	1.1 ± 0.25
16	3	9	0.88	1.6 ± 0.15
17	3	9	0.85	1.8 ± 0.2
18	3	9	0.73	∞
19	3	9	0.62	∞
20	3.5	9	1.24	0
21	3.5	9	1.13	0
22	3.5	9	1.02	0
23	3.5	9	0.88	0.7 ± 0.35
24	3.5	9	0.82	1.5 ± 0.25
25	3.5	9	0.71	∞
26	3.5	9	0.61	∞
27	3.5	9	0.5	∞

Table S1: Dimensional parameters corresponding to the experimental symbols presented in Fig. 2(a) of the main text.

Supplementary Note 3: Solutions for equilibria and phase transitions

The nondimensional equation for the pairwise force in the magnetic Cheerios system is given by

$$F = \frac{\mathcal{M}}{(l^*+1)^4} - e^{-2\sqrt{\mathrm{Bo}}\ l^*} \tag{S1}$$

which is equation (4) of the main text. We seek roots of Equation S1 corresponding to force equilibria. Introducing $L^* = -\frac{\sqrt{\mathrm{Bo}}}{2}(l^*+1)$, we have

$$\begin{aligned}
\mathcal{M}\left(-\frac{\sqrt{\mathrm{Bo}}}{2L^*}\right)^4 - e^{-2\sqrt{\mathrm{Bo}}\left[\left(-2L^*/\sqrt{\mathrm{Bo}}\right)-1\right]} &= 0 \\
\Rightarrow \mathcal{M}\left(-\frac{\sqrt{\mathrm{Bo}}}{2L^*}\right)^4 &= e^{4L^*}e^{2\sqrt{\mathrm{Bo}}} \\
\Rightarrow -\mathcal{M}^{1/4}\frac{\sqrt{\mathrm{Bo}}}{2}e^{-\sqrt{\mathrm{Bo}}/2} &= L^* e^{L^*}.
\end{aligned}$$

Solutions for L^* are given by the Lambert-W function $W_k\left(-\mathcal{M}^{1/4}\frac{\sqrt{\mathrm{Bo}}}{2}e^{-\sqrt{\mathrm{Bo}}/2}\right)$, where k represents the choice of branch. We then arrive at the expression for the equilibria l_k^*, given by

$$\Theta(\mathcal{M},\mathrm{Bo}) = -\mathcal{M}^{1/4}\frac{\sqrt{\mathrm{Bo}}}{2}\mathrm{e}^{-\sqrt{\mathrm{Bo}}/2} \tag{S2}$$

$$l_k^* = -\frac{2}{\sqrt{\mathrm{Bo}}}W_k(\Theta) - 1, \tag{S3}$$

where the argument of the Lambert-W function is defined as Θ. Lengths l_k^* given by Equation S3 correspond to force equilibria, and only positive values of l_k^* are physically realizable. The roots are ordered $-1 \leqslant l_0^* \leqslant l_{-1}^*$, with l_0^* corresponding to a stable equilibrium point and l_{-1}^* corresponding to an unstable equilibrium point.

To account for effects of steric interaction, we insist that only physically realizable separation distances are positive, i.e. $l^* \geqslant 0$. This can be modeled as an infinite step potential for $l^* < 0$. With this consideration, we can associate the roots l_k^* with the physically realizable equilibrium points $l_{\mathrm{eq}}^*, l_{\mathrm{cr}}^*$. First, we need to define the domain $\Omega(\mathcal{M},\mathrm{Bo})$ on which l_{cr}^* is defined, i.e. $l_{\mathrm{cr}}^* \geqslant 0$, given by

$$\Omega(\mathcal{M},\mathrm{Bo}) = (\mathcal{M} \leqslant 1) \text{ or } \left(\mathcal{M} \leqslant \frac{16}{\mathrm{Bo}^2}e^{-2\sqrt{\mathrm{Bo}}-4} \text{ and } \mathrm{Bo} \leqslant 4\right).$$

Then, the expressions for l_{cr}^* and l_{eq}^* are given by

$$\begin{aligned}
l_{\mathrm{cr}}^* &= -\frac{2}{\sqrt{\mathrm{Bo}}}W_{-1}(\Theta) - 1 \text{ on } \Omega \\
l_{\mathrm{eq}}^* &= \max\left(-\frac{2}{\sqrt{\mathrm{Bo}}}W_0(\Theta) - 1, 0\right) \text{ on } \Omega.
\end{aligned}$$

Outside of the domain Ω, no equilibrium points are physically realizable, and the system reverts to the *purely repulsive* phase.

To assess the stability of these critical points, we consider the derivative of S1 with respect to l^*:

$$\frac{dF}{dl^*} = \frac{-4\mathcal{M}}{(l^*+1)^5} + 2\sqrt{\mathrm{Bo}}\; e^{-2\sqrt{\mathrm{Bo}}\; l^*}. \tag{S4}$$

Evaluating this expression at the roots of S1 given by $l^* = l_k^*$, where $\frac{\mathcal{M}}{(l_k^*+1)^4} = e^{-2\sqrt{\mathrm{Bo}}\; l_k^*}$, S4 becomes

$$\left.\frac{dF}{dl^*}\right|_{l^*=l_k^*} = \left(\frac{-4}{l_k^*+1} + 2\sqrt{\mathrm{Bo}}\right)\left(e^{-2\sqrt{\mathrm{Bo}}\; l_k^*}\right). \tag{S5}$$

The second term on the right-hand side is always positive, and the first term on right-hand side is positive when $l_k^* > l_{tr}^*$ and negative when $l_k^* < l_{tr}^*$, where $l_{tr}^* = \frac{2}{\sqrt{\mathrm{Bo}}} - 1$ represents a branch point of $\mathcal{W}(\Theta)$. This is equivalent to the choice of branch, implying that l_{eq}^* is always stable and l_{cr}^* is always unstable.

The definition of each phase boundary can be clarified through the value of the argument $\Theta(\mathcal{M}, \mathrm{Bo})$, as seen in Figure S2.

In the definition of Θ in Equation S2, roots of $\mathcal{M}$ and Bo are taken to be positive, and thus the argument $\Theta \leqslant 0$ for all physical configurations.

When $-1/e < \Theta < 0$, there are two real solutions for l_k^*. The *mermaid* regime holds when both of these solutions are positive, and the *Cheerios* regime holds when only one of these solutions is positive. If both roots are negative, there are no physically realizable equilibria, and the *purely repulsive* regime holds.

Similarly, for $\Theta < -1/e$, there are no real solutions for l_k^*, also corresponding to the *purely repulsive* case with no equilibrium points.

When $\Theta = -1/e$, there is a single equilibrium point $l_{\mathrm{tr}}^* = \frac{2}{\sqrt{\mathrm{Bo}}} - 1$, and this value for the argument defines the transition boundary between the *mermaid* and *purely repulsive* phases for $l^* > 0$. This boundary is defined by

$$\mathcal{M} = \frac{16}{\mathrm{Bo}^2} e^{2\sqrt{\mathrm{Bo}}-4} \quad \text{and} \quad \mathrm{Bo} \leqslant 4.$$

When $l_{\mathrm{tr}}^* = 0$, occurring for values $\mathrm{Bo} = 4$ and $\mathcal{M} = 1$, this system reaches a triple point between the *mermaid*, *Cheerios*, and *purely repulsive* phases. This is indicated by a star in the phase plot of Figure S2.

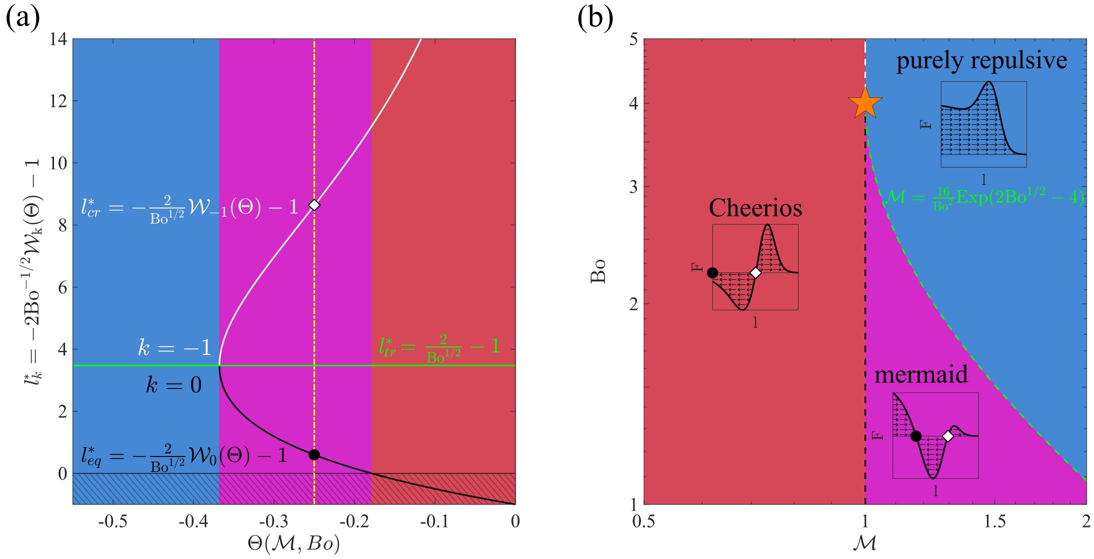

Figure S2: Panel (a) shows the branches of the expression for the equilibrium lengths and corresponding phases. The hatched region $l^*_k < 0$ indicates locations where the roots l^*_k are not physically attainable. The white curve represents the $k = -1$ branch, and the black curve represents the $k = 0$ branch. The green line represents the transitional length l^*_{tr}. For a certain value of the argument Θ, indicated by the dashed yellow line, the intersections with the black and white curves represent the two solutions l^*_0, l^*_{-1}. Panel (b) shows the corresponding phases as a function of $\mathcal{M}$, Bo. The triple point, located at $\mathcal{M} = 1, \mathrm{Bo} = 4$ is indicated by a gold star. The dashed green line represents the transition between the *mermaid* and *purely repulsive* phases. Inset plots show examples of force as a function of length in each phase. In both panels, the *purely repulsive* phase is colored blue, the *mermaid* phase is colored violet, and the *Cheerios* phase is colored red. l^*_{eq} is represented by a black circle, and l^*_{cr} is represented by a white diamond.
