# Supplementary material for: Interactions and pattern formation in a macroscopic magnetocapillary SALR system of mermaid cereal
Source: Nat Commun. 2024 Jun 27;15:5466. doi: 10.1038/s41467-024-49754-4 (PMC11211465; doi:10.1038/s41467-024-49754-4)
Supplement: Supplementary file 3 — Description of Additional Supplementary Files [file 41467_2024_49754_MOESM3_ESM.docx]

File Name: Supplementary Video 1

Description: Interaction of two disks in the mermaid regime. Associated with Figure 1 of the main text.

File Name: Supplementary Video 2

Description: Interaction of two disks in the fully repulsive regime. Associated with Figure 1 of the main text.

File Name: Supplementary Video 3

Description: Pattern formation in 2D with disks in the fully repulsive regime. Associated with Figure 2(b) of the main text.

File Name: Supplementary Video 4

Description: Pattern formation in 2D with disks in the mermaid regime. Associated with Figure 2(c) of the main text.

File Name: Supplementary Video 5

Description: External control of equilibrium pattern. Associated with Figure 4 of the main text.
